# Liquid Droplet Microresonators

**DOI:** 10.3390/s19030473

**Published:** 2019-01-24

**Authors:** Antonio Giorgini, Saverio Avino, Pietro Malara, Paolo De Natale, Gianluca Gagliardi

**Affiliations:** 1 Consiglio Nazionale delle Ricerche, Istituto Nazionale di Ottica (INO), via Campi Flegrei 34—Comprensorio A. Olivetti, 80078 Pozzuoli (Na), Italy; antonio.giorgini@ino.it (A.G.); saverio.avino@ino.it (S.A.); pietro.malara@ino.it (P.M.); 2 Consiglio Nazionale delle Ricerche, Istituto Nazionale di Ottica (INO), Largo E. Fermi 6—50125 Firenze, Italy; paolo.denatale@ino.it

**Keywords:** whispering gallery modes, droplet micro-cavity, free-space laser excitation, optical Q-factor, cavity ring-down spectroscopy, cavity optomechanics

## Abstract

We provide here an overview of passive optical micro-cavities made of droplets in the liquid phase. We focus on resonators that are naturally created and suspended under gravity thanks to interfacial forces, illustrating simple ways to excite whispering-gallery modes in various slow-evaporation liquids using free-space optics. Similar to solid resonators, frequency locking of near-infrared and visible lasers to resonant modes is performed exploiting either phase-sensitive detection of the leakage cavity field or multiple interference between whispering-gallery modes in the scattered light. As opposed to conventional micro-cavity sensors, each droplet acts simultaneously as the sensor and the sample, whereby the internal light can detect dissolved compounds and particles. Optical quality factors up to 107–108 are observed in liquid-polymer droplets through photon lifetime measurements. First attempts in using single water droplets are also reported. These achievements point out their huge potential for direct spectroscopy and bio-chemical sensing in liquid environments. Finally, the first experiments of cavity optomechanics with surface acoustic waves in nanolitre droplets are presented. The possibility to perform studies of viscous-elastic properties points to a new paradigm: a droplet device as an opto-fluid-mechanics laboratory on table-top scale under controlled environmental conditions.

## 1. Introduction

**Figure 1:**
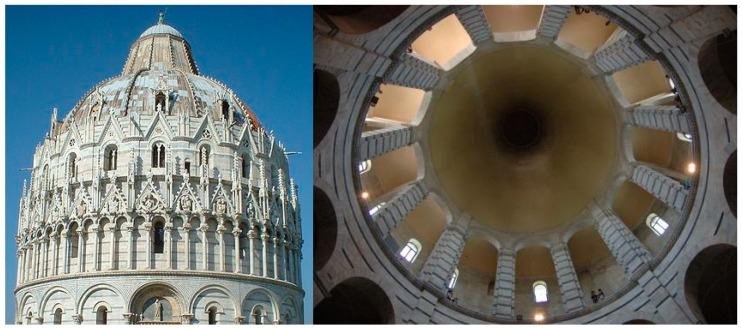
Photos of Pisa baptistery’s dome (**left**) and of its internal gallery structure (**right**).

Resonant phenomena in cavities, be they optical, acoustic or mechanical, are basically dependent on the geometrical features of the supporting structure, i.e., on size and shape. Accordingly, such resonances are often termed morphology-dependent resonances (MDRs). One of the most famous examples of MDRs, in the acoustic domain, is that of whispering gallery modes (WGMs). First explained by Lord Rayleigh, these modes correspond to traveling pressure waves guided around a closed concave surface, such as the renowned whispering gallery of St. Paul’s Cathedral in London [1] or Pisa baptistery [2] (see Figure 1). From a geometric perspective, such bound modes are guided by repeated reflections, which, in absence of absorption and scattering, would continue ad infinitum. Within a wave description, however, losses through the surface manifest via tunneling or frustration such that the mode experiences a decay in its amplitude, i.e., a finite lifetime. WGMs are actually a subclass of MDRs characterized by high mechanical quality (low losses) and shallow nature. There is a strict analogy between such sound modes and electromagnetic modes occurring in dielectric microresonators, where similar phenomena have been observed since the beginning of the 20th century [3]. Under proper conditions, these two types of waves may even interact and exchange energy within the resonant structure. Indeed, the interplay between electromagnetic radiation and mechanical motion in solid dielectrics via ponderomotive forces or sound scattering has been extensively investigated, also in recent studies [4].

Spheres, discs, and rings represent the simplest optical whispering-gallery resonator geometries and have thus received much attention in the literature over the years. In WGMs, light circulates for a long time along equatorial trajectories close to the surface before it is scattered or absorbed [5]. Thanks to low optical losses of silica and other optically-transparent materials, narrow resonances with quality factors (Q-factors) in excess of 10^9^ may be excited in the visible and near-infrared spectral regions [6,7,8]. A multitude of applications have been demonstrated over the past several years, such as wavelength-multiplexing [9], bio-chemical sensing [10,11], optical frequency comb generation [12,13], and cavity opto-mechanics [14]. Effective uses of microspheres and microtoroids as bio-chemical sensors have been demonstrated, in most cases, measuring the fluorescence [15] and the near-resonance shift [16,17] or measuring the Q-factor for gas and nanoparticle detection [18,19,20]. Indeed, if resonant light interacts with a molecular compound in proximity of its absorption features or with a scattering object, the lifetime of photons in the cavity may be reduced. However, the vast majority of previous works have been carried out on solid resonators, but these resonators are not readily adaptable to probe molecules in liquid samples while WGM detection schemes based on tapered-fibers or prism coupling configurations are not well suited to integration within chemical analyzers and microfluidic systems. These aspects are crucial for bio-sensing applications. Detection in liquids with WGMs was achieved using low-Q micro-disk resonators based on silicon-on-insulator (SOI) combined with microfluidics [21] or, in rare cases, using silica micro-resonators totally immersed in water, kept in a closed and controlled environment, where optical resonances were excited by a wavelength-swept laser [22]. In the latter case, the surrounding liquid typically causes a dramatic decrease of the cavity optical quality. In addition, despite the impressive Q-factor of crystalline solid WGM resonators, most of the light is confined inside its dielectric medium whereas only the weak evanescent tail interacts with the external sample, thereby significantly decreasing the cavity enhancement in sensing and spectroscopy applications [18]. In this regard, liquid resonators emerged recently as a new frontier. Indeed, there have been several attempts to integrate a liquid sample within an optical resonator, harnessing optical WGMs of microcapillaries and microbubbles [23,24]. A different approach consists in using spheroidal optical cavities directly made out of liquid, without resorting to external shells.

Here, we report on recently-developed schemes that enable passive WGM excitation in single, stable droplets and show how these schemes, on the one hand, have progressively led to experiments similar to those performed with solid microresonators and, on the other hand, have provided new pathways for optical sensing, opto-fluidics and opto-mechanics directly performed in liquids.

## 2. Droplets as Optical Microcavities

Natural surface tension shapes droplets into almost-perfect resonators. Indeed, droplets laying on a flat surface, blown through a hollow capillary or suspended by a thin wire all have excellent features in terms of optical quality, thanks to surface tension that creates an atomic-scale smooth surface. In addition, gravity action is negligible compared to interfacial forces if the involved liquid mass is small. The relevant quantity, in this case, is the capillary length $\Lambda =\frac{\gamma }{\rho g}$, where γ and ρ are the surface tension and mass density of the fluid. If the sample size does not exceed Λ, the drop remains stable and takes a nearly spherical geometry.

A fundamental parameter that is conventionally used to quantify the quality of any optical resonator is the Q-factor [25]

(1) $$Q=\omega_{0}\tau$$

where $\omega_{0}$ is the optical (angular) frequency and τ is the cavity photon lifetime, i.e., the time effectively spent by light within the cavity on resonance. In the simple case where material absorption is the only loss channel, Equation (1) reduces to

(2) $$Q_{\mathrm{abs}}=\frac{2\pi n}{\alpha \lambda }$$

where n and α are the liquid refractive index and absorption coefficient (expressed in m-1) at wavelength λ, respectively. In the case of droplet cavities, for most liquid media, typically ranges between 1.3 and 1.5 whereas α strongly depends on the wavelength. The visible region is the spectral interval where pure liquids have their maximum transparency and thus the highest Q-factor is expected for a droplet. This is the case of water, for example, which shows transmission windows around 640 nm and 470 nm [26]. Using one among the various experimental estimates of water absorption coefficient, Equation (2) returns a value ranging between 10^8^ and 10^9^, i.e., close to that obtained in recent works with solid glass microresonators.

**Figure 2:**
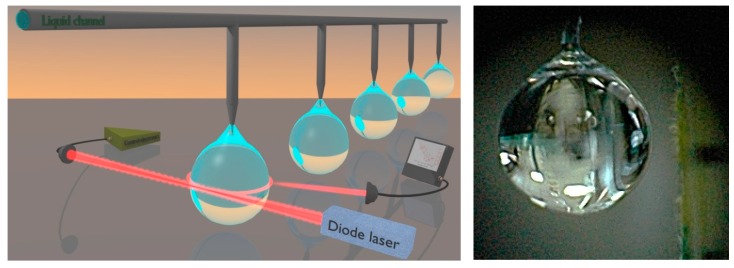
**Left**: 3D rendering of the optical scheme for excitation of a droplet cavity by a free-space laser beam (Reproduced with permission from [27]). **Right**: photograph of a liquid-paraffin droplet (~1.5 mm diam) hanging from the tip of a silica fiber with acrylic coating (250-μm outer diam).

A droplet offers a number of benefits as a WGM sensor. First of all, the analyte can be incorporated into the (host) liquid material, such that the droplet serves as the sensor and the sample at the same time (see Figure 2). In this way, analytes and/or particles interact with the most intense fraction of the WGM, inside the cavity, thus leading to higher detection sensitivity. Droplet resonators can be easily created without the need for complex fabrication procedures, whilst surface tension ensures nearly atomic-scale smoothness, reducing optical scattering-losses. Surface scattering losses are ultimately influenced by thermal fluctuations. Moreover, liquid droplets lend themselves to integration with microfluidic delivery and analysis set-ups. On the other hand, compared to solids, a droplet is much softer and thus undergoes more pronounced opto-mechanical effects. Strong convection, nonlinear transport effects as well as acoustic excitation phenomena can be observed.

In the following, we illustrate how to build an effective optical excitation scheme and how to extend spectroscopic techniques to liquid microresonators.

### 2.1. Whispering Gallery Mode Excitation in Liquids

In the pictorial view of Figure 2, a liquid droplet, vertically suspended by the tip of a silica fiber, serves as the optical cavity interrogated by a laser [27]. A very simple way to create and manipulate a droplet consists of attaching a sample to the tip of a thin wire, with volumes ranging from ~0.1 to 3 μL as far as liquid surface tension and liquid–solid cohesion counteract the gravity force. For instance, this is feasible using a glass capillary or a silica fiber that ensures a stable position and alignment of the cavity at the same time. A syringe can also be used to control the volume and directly inject contaminants or particles into the liquid drop. The droplet holder may be fixed directly on the optical table without the need for any closed chamber around. Droplet cavities made of water-based samples can be suspended, achieving similar or higher Q-factors but paraffin, PEG, silicone oil etc. have been preferred in preliminary studies owing to their low evaporation rate.

The whispering-gallery modes may be excited by focusing a free-space beam at the droplet rim, tangential to the surface, without using any special coupling device. In this configuration, the focus optimal position is slightly inside the surface edge and the cavity is undercoupled, typically with 5–10% of the incident power being effectively injected into low-order WGMs [27]. The overall coupling efficiency also depends on the microresonator Q (and thus on the excitation wavelength) as the laser-emission spectral overlap with the cavity mode decreases with decreasing the mode linewidth [25]. A careful alignment of the droplet into the laser beam requires an xyz micrometric translation stage while microscope objectives are adopted to focus the laser radiation and collect the directly transmitted and scattered beams by the droplet on separate photodetectors. This alignment is easily reproducible and gives always similar mode patterns, even for different sizes. Thanks to the natural liquid damping, acoustic noise and air flows in the laboratory do not seriously perturb the cavity.

**Figure 3:**
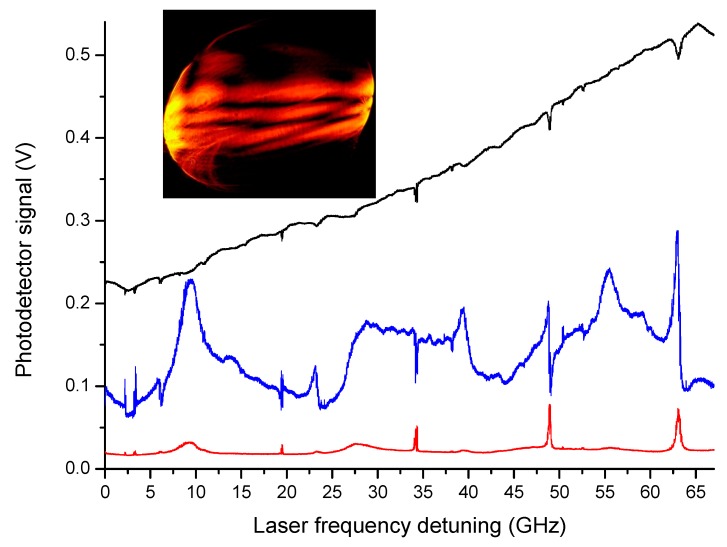
Whispering gallery mode (WGM) resonances recorded from the transmission (black line), back-scattering (red) and side-scattering (blue) of a pendant liquid paraffin droplet excited by a 640-nm laser beam with incident power of 5 mW. The observed coupling efficiency for the central mode is about 6%. In the inset, a camera view of the side scattered light evidences the presence of different WGMs.

Basically, the main physical mechanisms responsible for optical coupling from free-space are believed to be surface scattering [28,29] and chaos-assisted tunneling [30], whereas evanescent-wave matching is not relevant due to the small droplet curvature (for radii >50 μm) [31,32]. As remarked in ref. [33], van de Hulst’s localization principle fails in the case of a droplet with surface irregularities. Thus, weak distortions caused by thermal capillary waves induce angular-momentum coupling of partially-scattered light waves to the resonator WGMs achieving the highest excitation efficiency for a beam impact parameter slightly smaller than the drop radius [33,34], in agreement with experimental findings. If a current scan is sent to the laser, several WGM spectra can be observed in a few-ms time. Figure 3 shows 5 narrow resonances detected for a liquid paraffin droplet (300 μm radius), over a cavity free-spectral-range, using a 640-nm diode laser as a light source. Here, peaks appear in the scattering while corresponding dips appear in the transmission. The scattered light is collected both in a quasi-counterpropagating direction (passing backwards through the focusing objective) and at a 90-degree angle with respect to the incident beam (side-scattering), observing different lineshapes around the resonances. These spectra are highly repeatable and the alignment of the droplet is not critical to the quality factor as opposed to tapered-fiber or prism coupling schemes [34]. The Q-factors of the droplet cavities have been characterized with cavity ring-down spectroscopy [27,35], as shown in the next section. At a wavelength of 640 nm, the longest measured lifetime was (6.35 ± 0.05) ns, corresponding to a Q-factor of (1.87 ± 0.01) × 10^7^.

Due to free-space illumination, a number of resonances are simultaneously excited in the liquid cavity, being likely associated to WGMs of low and high radial orders with narrower and wider lineshapes, respectively. In addition, the ellipticity of suspended droplets may split WGMs among different azimuthal orders. A comprehensive theoretical description of scattering efficiency and internal intensities for a dielectric sphere can be found in [36], reproducing quite well the experimentally observed spectra. In the case of liquid droplets, the WGM spectrum is strongly influenced by the suspension tool (capillary, wire or needle) and the axially-symmetric deformation caused by gravity. Basically, defining an ellipticity parameter *e* = (*r_p_* − *r_e_*)/*R* (where *r_p_* and *r_e_* are the radii in the polar and equatorial direction, respectively, and R = (*r_p_* + 2*r_e_*)/3)), which quantifies the deviation of droplets from a perfect spherical geometry, each equatorial mode splits over separate modes whose resonance frequencies obey the following relation [37,38]:

(3) $$\nu (m)=\nu_{0}\left[1-\frac{e}{6}\left(1-\frac{3m^{2}}{l(l+1)}\right)\right]$$

where *m* and *l* are the azimuthal mode number and the angular momentum number of the WGM, respectively (*m* = [−*l*, *l*]). The natural degeneracy of lowest order (longest lifetime) modes is lifted since an oblate or prolate ellipsoid shape allows additional equatorial trajectories (resonances) having slightly different energies (frequencies). From Equation (3), the frequency separation between different azimuthal modes, corresponding to *l* = *l*_max_ and *n* = 1 (*n* being the radial mode number), is given by

(4) $$\Delta \nu (l)=\nu_{0}e/l$$

From the non-degenerate multiplet spectrum of Figure 3, in a given WGM condition, the frequency separation Δν(l) between adjacent azimuthal modes is found to be ~11.4 GHz. Inserting this Δν(l) value in Equation (4) and considering, for the lowest-order WGM, that $l_{max}\approx 7100$ ($l_{max}\approx 2\pi NR/\lambda$ for a *spherical* resonator with 1-mm diameter and refractive-index *N* = 1.45, at a 640-nm wavelength), an experimental ellipticity *e* ~ 0.17 is retrieved, which agrees well with that observed from direct camera images of the selected droplet.

**Figure 4:**
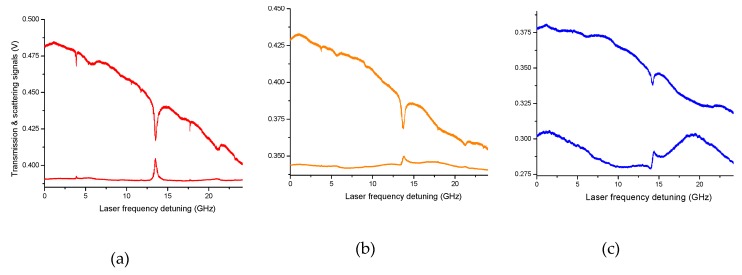
Fano-resonance behavior observed varying the distance between the laser beam and the droplet. The relative distance is decreased by ~1.6 μm (**b**) and 2.8 μm (**c**) from the WGM optimal coupling position (**a**).

By a closer inspection of the resonances shown in Figure 3, one notes asymmetric profiles in the side-scattered signals, which likely originate from the interference between modes of different orders and spatial distribution. In particular, Fano resonances show up at frequency positions where a low-Q WGM and a high-Q WGM interact with proper relative detuning [30,39]. In Figure 4, this phenomenon is investigated in detail. The transmitted and side-scattered light powers are monitored around a specific cavity resonance while the distance between the laser-beam focal point and the droplet is finely varied by a piezo-actuated translator (20-nm resolution). Starting from optimal coupling to the shallowest WGMs, with the beam impinging slightly inside the surface, the distance was decreased further in order to change the amount of power injected into higher-order (lower-Q) resonances. As a result, both in transmitted and scattered intensities, a new, broader mode emerges from the background and the narrower mode takes an asymmetric spectral profile. When the distance is decreased by ~2.8 μm from the initial position, we observe a typical Fano dispersive lineshape that is particularly pronounced on the far-field side-scattered light.

### 2.2. Laser Frequency Locking to Optical Resonances

**Figure 5:**
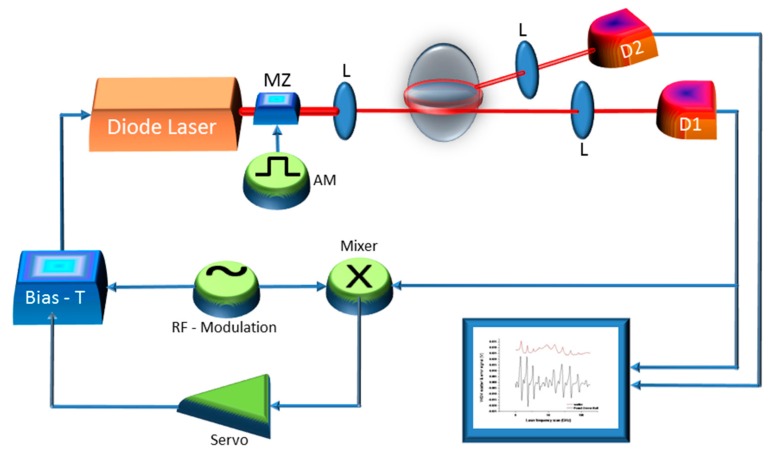
Laser light coupling, detection and frequency locking scheme. D1, D2, photodetectors; RF, radio-frequency; AM, amplitude modulation; MZ, Mach–Zehnder modulator; L, lens (Reproduced with permission from [27]).

**Figure 6:**
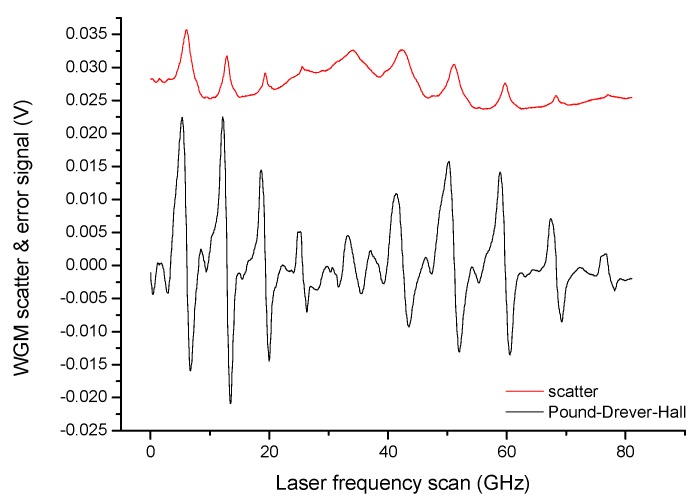
Pound–Drever–Hall (PDH) error signals (black line) generated after demodulation of the directly transmitted power with sidebands at about 1 GHz (paraffin oil droplet) using a diode laser emitting around 1560 nm (incident power 2 mW). The WGM scatter spectrum (red line) shows peaks that are centered exactly at the PDH signal zero crossing points (All plots are reproduced with permission plots from [27]).

Widely-tunable lasers emitting around different wavelengths, centered from the near-infrared to the visible spectral region, have been used to interrogate liquid droplet microresonators. In particular, using distributed-feedback (DFB) diode lasers is particularly convenient as entire free-spectral-ranges between fundamental WGMs could be observed through fast and deep scans of the injection current (~100 GHz). However, when the WGM linewidth becomes comparable or narrower than the laser emission width, the use of a higher-coherence laser becomes necessary. The laser optical frequency can also be ‘locked’ at the peak of isolated resonances by means of active feedback systems based on different techniques. For this purpose, the Pound–Drever–Hall (PDH) technique proved particularly effective for high-bandwidth laser stabilization on cavity spectral features with various schemes [40]. In the system shown, in Figure 5, for example, a DFB diode laser emitting in the near-infrared (1560 nm) is phase modulated at high frequency (typically 1–1000 MHz, depending on the cavity linewidth), through the laser anode or an external phase modulator, to generate symmetric sidebands around the optical carrier. Then, the cavity leakage field, provided by the transmitted beam, is detected by a fast InGaAs/Si photodiode whose output signal beats with the modulation signal in a radio-frequency mixer. The mixer then yields a dispersive-like signal crossing zero at the resonance (see Figure 6) that serves as the error signal of a proportional-integrative electronic loop (servo-amplifier) acting on the laser current. This scheme proved very stable and effective for WGM tracking as well as cavity ring-down spectroscopy in droplet microcavities [27].

Actually, it has also been shown that a suitable frequency discriminator for laser-to-microcavity locking can be derived from the interference of multiple cavity modes or, in the free-space excited droplets, from the scattered WGMs [41]. Thus, the light reflected by the cavity contains the interference pattern among wavefronts of various modes (see inset of Figure 3). In a free-space focused-beam configuration to excite a droplet cavity, this condition is well satisfied as the scattered light provides the overlap between WGMs of different orders. In this case, the optical set-up is similar to that shown in Figure 5 but without modulation/demodulation electronics.

**Figure 7:**
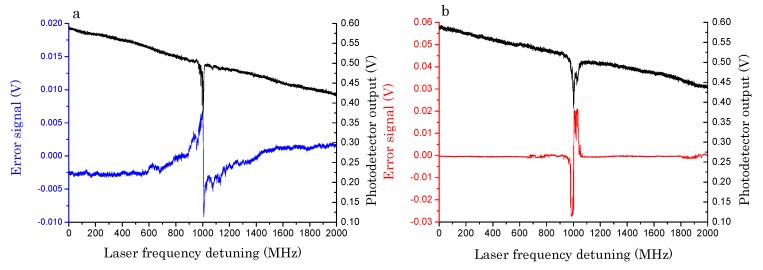
WGM resonance along a laser frequency sweep on the directly-transmitted beam (black line) and the corresponding error signal generated by (**a**) spatial interference on the back-scattered light (blue line) and (**b**) Pound–Drever–Hall technique (red line) (All plots are reproduced with permission plots from [41]).

Thanks to the free-space excitation, a superposition of WGMs with different orders and amplitude profiles, each one having its own phase, is generated. By spatial selection of the cavity scattered light, the interference term between a narrow WGM and a secondary mode is recovered, the latter acting as a phase reference. This selection can be carried out by a diaphragm, located just in front of the photodetector PD2, whose position must be carefully adjusted until an odd-symmetrical profile is obtained [41]. The resulting signal exhibits a dispersive lineshape with a symmetry point located at the main resonance center and an amplitude proportional to the imaginary component of the resonant optical field. PD2 output is sent to a summing amplifier where a variable offset is subtracted when the laser is far from the WGM resonance. After subtraction, the signal has a zero-crossing at the resonance center and can be used as the error of an electronic stabilization circuit. The spatial-mode interference (SMI) signal of a high-Q WGM is shown in Figure 7a and compared with the error signal obtained with a PDH technique on the same resonance, shown in Figure 7b. The latter is obtained by superimposing sidebands at 70 MHz to the laser carrier by direct phase modulation via its bias-tee input and demodulating the PD1 signal in a RF double-balanced mixer [27]. It is worth noting that both discriminators exhibit a sharp transition in the vicinity of the resonance, with a signal-to-noise ratio (S/N) of ~110 and 600 for the SMI and PDH schemes, respectively, but their behavior far from the center is rather different: in the SMI scheme, the signal wings decay very smoothly, as already observed with locking techniques based on polarization spectroscopy [42]. That allows a faster recovery of the locking condition, thereby making the SMI method intrinsically more robust. In order to lock on the WGM peak, the error signals are sent to a proportional-integrative servo amplifier whose output is fed back to the laser current generator. Once the loop is closed and the servo gain is increased up to an optimal value, the laser settles on the maximum resonance transmission without suffering from acoustic disturbances or thermal drifts. The system stability was tested over 30 min without observing significant degradation of the locking efficiency. From the fast Fourier transform (FFT) of the error signal, in the SMI and PDH schemes, we found that their locking performance, using the same servo circuit, is substantially the same: their frequency response has a unity-gain bandwidth of about 5.4 kHz and maximum noise suppression ~ 40 dB at low frequencies [41]. In addition, the SMI is simple and cheap compared to other approaches as it does not require polarization elements, radio-frequency components, very-fast photodetectors, or any laser modulation. With either the above schemes, the laser can be tightly frequency stabilized on a droplet WGM so that the servo output provides a real-time monitor that tracks the resonance shifts from DC up to the locking bandwidth.

## 3. Droplet Q-Factor and Photon Lifetime

### 3.1. Effects of Absorption, Scattering and Thermodynamic Noise

The Q-factor of a droplet microresonator for a specific WGM, with optical frequency ν, can be expressed in terms of the different loss channels that contribute to reduce the overall photon lifetime τ, that is

(5) $$\frac{1}{Q}=\frac{1}{Q_{a}}+\frac{1}{Q_{r}}+\frac{1}{Q_{ss}}+\frac{1}{Q_{t}}$$

where *Q* = 2*πντ* and *Q*_a_, *Q*_r_, *Q_ss_*, *Q_t_* are the contributions of material absorption, radiative loss, surface scattering loss and thermal shape distortions, respectively. A relative decay rate can be associated to each of the above terms. As remarked in the previous section, in microresonators with radii of the order of a few hundred µm, the radiative loss is negligible compared to the surface scattering loss, which in fact is the main in-out coupling mechanism. Hence, the other terms related to absorption and thermal noise rule the quality budget of liquid microresonators. In particular, shape distortions due to thermally-induced capillary waves in the liquid have clearly been evidenced by theoretical models as potential limitation to the observed Q-factor [43]. These distortions modulate the splitting of quasi-degenerate azimuthal modes via a dynamic change in droplet eccentricity, resulting in an effective WGM line broadening. The associated Q-factor reduction, considering the dependence of the azimuthal mode frequency on the droplet eccentricity for a WGM with the lowest radial order and maximum angular momentum (l≈2πna/λ), can be derived from [44]

(6) $$Q_{t}≅\frac{2a}{\delta }$$

where δ is the root-mean-square amplitude of the thermally-induced droplet fluctuations, given in [45].

The theoretical limit set by thermal fluctuations for a droplet with *a* = 450 µm would be $Q_{t}^{*}$ = 1.6 × 10^6^ (with paraffin and mineral oils). In the particular case where the only contribution is material absorption, using *n* = 1.47 and *α* ≈ 0.7 m^−1^ [46,47] in Equation (2), one gets $Q_{a}$ ≅ 2 × 10^7^. Heavy water (D_2_O) would exhibit even higher absorption-limited Q, having *α* ≈ 0.02 m^−1^ at 640 nm [26].

Basically, the Q-factor of an optical cavity can be measured in different ways. The most popular is a spectroscopic measurement of the WGM resonance linewidth. However, this measurement is not always accurate as it requires a non-linear fit of the WGM resonance profile that, for high Q-factors, is affected by laser-cavity frequency fluctuations, amplitude noise, acoustic noise, and photothermal effect, which all change significantly with the observation timescale [25,48,49]. A time-domain measurement of photon lifetime provides instead a direct, reliable, high-sensitivity estimate of the intra-cavity loss. This can be done by cavity ring-down spectroscopy (CRDS) technique [25,27]. Unfortunately, microresonators typically exhibit very short lifetimes (1–10 ns), thus making ring-down measurements technically challenging even for very fast opto-electronic components. Also, it only provides the loaded Q-factor value. A good alternative is the so-called rapidly-swept CRDS, where the laser is not constantly resonant but is scanned through the resonance faster than the cavity build-up [25,50], thus generating a temporal beat-signal between photons entering the cavity and those trapped into it. In this way, a chirped ringing response emerges on the transmission carrying information on the scan rate, the intrinsic cavity lifetime and the coupling loss rate [51]. The mathematical function that represents the microresonator transmission in a non-stationary condition is given by T $={\left|\frac{E_{out}}{E_{in}}\right|}^{2}$ with $E_{out}$ and $E_{in}$ the output and input cavity fields, respectively, which are related each other by [52]

(7) $$E_{out}=-E_{in}+\sqrt{\frac{2}{\tau_{c}}}v\left(t\right)$$

where

(8) v(t)=2τcEinexp(jω0−tτ)[f(t)−f(0)+τ1+j(ωi−ω0)τ]

(9) $$f\left(t\right)=-\sqrt{\frac{j\pi }{2V_{s}}}\exp\left[-\frac{j{\left(\omega_{i}-\omega_{0}-j/\tau \right)}^{2}}{2V_{s}}\right]erf\left(\frac{\frac{j}{\tau }\;+\;\omega_{i}-\omega_{0}-V_{s}t}{\sqrt{2jV_{s}}}\right)$$

*erf*(*z*) with z ∈ C denotes the complex error function, 1τ=1τc+1τ0 is the loaded photon lifetime, $\tau_{c}$ is the loss rate associated with the out-coupling and $\tau_{0}$ is the intrinsic photon lifetime.

**Figure 8:**
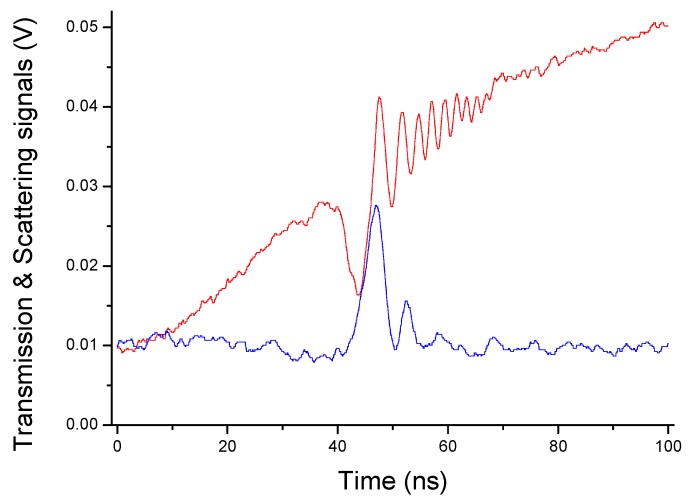
Ringing lineshapes observed during a fast laser-frequency sweep through the narrowest WGM shown in Figure 3, for a paraffin oil droplet (red line is recorded from direct transmission, blue line from back-scattering).

In Figure 8, we show the ringing profile, recorded with ultra-segmentation mode and averaged over several waveforms for the resonance excited after ~34 GHz along the scan, at the center of Figure 3. A nonlinear fit with Equation (7) yields a lifetime $\tau^{\prime }$ = 4.8 ± 0.1 ns for this mode. The resulting Q values are one order of magnitude higher than the thermal-noise bound $Q_{t}^{*}$. Also, we repeated the same measurements varying the beam-droplet distance without finding any significant change in lifetimes.

Therefore, the large measured Q-factors are beyond the expectations based on thermal fluctuations. To verify this conclusion, we suspended four paraffin-oil droplets with radii varying from 350 μm to 600 μm, and performed subsequent measurements with the time-ringing technique on the central mode. The decay times fall between 4.43 ± 0.04 ns and 4.66 ± 0.05 ns, i.e., Q-factors between (1.30 ± 0.01) × 10^7^ and (1.37 ± 0.01) × 10^7^. From this measurement set, we note that the lifetimes remain constant with the drop radius within 2σ, even when the size is doubled. The τ value changes significantly if a different liquid is used instead (5.44 ± 0.05 ns for silicone oil, corresponding to Q = (1.60 ± 0.01) × 10^7^,). This argument leads us to conclude that the quality factor is still affected by intra-cavity absorption loss. Also it confirms that thermal noise is not the actual limit since the dependence on the droplet radius expressed in Equation (6) is not found in the experiment. Interestingly, the quality factor *Q* = *ν*/*Δν* retrieved from the linewidth *Δν* of WGM resonances in a stationary condition, observed on a much lower scan rate (0.5-ms transit through resonance), is ~3 × 10^6^ [35]. This value is close to the limit predicted by Equations (2) and (3). The discrepancy with the ultrafast time-domain measurements is might be due to thermally-induced shape distortions on intermediate timescales (50–0.5 μs).

It is worth noting that all the fits provide an out-coupling lifetime $\tau_{c}$ ≈ 7 µs, i.e., >1500 times larger than the intrinsic lifetime $\tau_{0}$, which points out that the liquid cavity does not suffer from any loading effect with free-space excitation. We note that the decay rate $1/\tau_{c}$ is a direct estimate of the out-coupling efficiency, i.e., the scattering loss. Surface scattering can be estimated from [8]: using an expression tested for solid microresonators, one obtains a $Q_{ss}$ ~ 2 × 10^10^ corresponding to a lifetime $Q_{ss}/2\pi \nu$ ~ 6.9 µs, in satisfactory agreement with the $\tau_{c}$ retrieved from ringing measurements.

Thus, despite theoretical models that would indicate thermo-mechanical noise as the fundamental limit, experimental results indicate that, on a time-scale shorter than thermal fluctuations, the Q-factor of free-space coupled liquid microresonators is still limited by material absorption, which points to a quality factor >10^8^ for high-transparency liquids. When the laser passage through resonance is observed along a timescale shorter than that of typical cavity distortions, the ringing decay time discloses the intrinsic cavity lifetime unaffected by thermal and mechanical noise.

**Figure 9:**
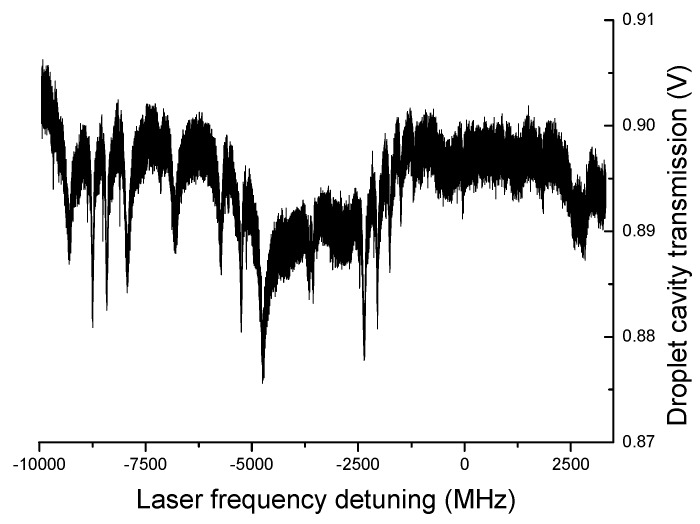
WGMs recorded on the transmission of a pure H_2_O droplet using a laser at 475 nm.

Finally, we excited optical resonances of H_2_O and D_2_O droplets at even shorter wavelengths, where water and most liquids exhibit their maximum transmission. For this purpose, we used an extended-cavity diode laser emitting at 475 nm with a spectral linewidth of 100 kHz. In Figure 9, an example of such recordings is shown for a H_2_O droplet suspended by a thin silica wire and excited with the free-space coupling scheme. Here, the cavity Q-factor approaches 10^9^ and, as expected, is mainly limited by water absorption (~2 × 10^−2^ m^−1^ [26]).

### 3.2. Liquid Contamination

When a near-infrared or visible laser is frequency locked to a droplet resonance, the laser tracks any WGM shift whether it is due to droplet vibrations, deformation or possible chemical reactions occurring on a time scale within the locking-loop bandwidth (10 kHz). In ref. [27], a diode laser emitting at 1560 nm was frequency locked to a WGM resonance by the Pound–Drever–Hall (PDH) technique [41] while cavity ring-down spectroscopy (CRDS) was performed to measure its Q-factor.

**Figure 10:**
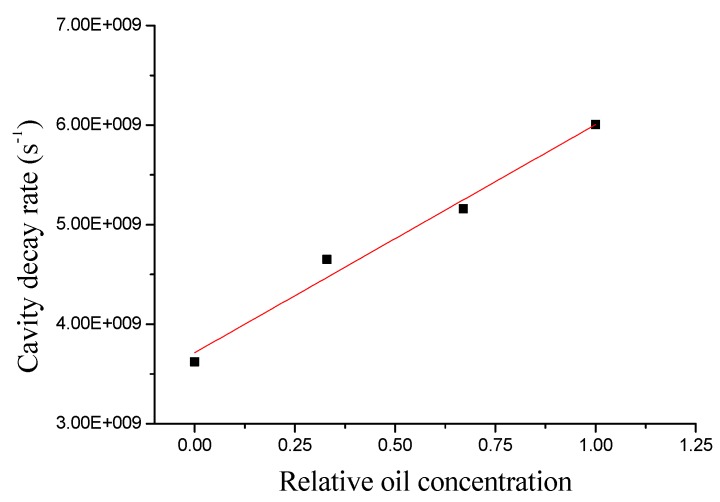
Photon decay rate for increasing olive-oil/seed-oil concentration levels (Reproduced with permission from [27]).

As a proof-of-concept, different liquids having different absorption at the laser wavelength were used, thus being distinguishable from the difference in the corresponding quality factors. Using mixtures of seed oil and olive oil, their absorbance was retrieved via CRDS measurements. In Figure 10, the measured cavity decay rate shows a linear dependence on the relative oil concentration. The slope agrees well with the measured ratio between the absorption coefficients of the two oils. Thus, the chemical purity of oils can be characterized through the optical quality of a drop.

### 3.3. Particle Detection

The laser-to-cavity lock scheme also provides a real-time monitor of the adhesion of small objects, even with sub-wavelength dimensions, or upon their passage through the equatorial direction. Indeed, optical losses are induced by particle scattering and can be observed, as recently proposed in ref. [20], from WGM line broadening. In our system, CRDS can be more effectively used to detect metallic or dielectric nanoparticles without off-line fitting of the WGM resonance profile. As opposed to direct measurements of the WGM spectrum, CRDS is extremely fast and, in principle, immune from laser amplitude and frequency fluctuations. Furthermore, the lock to a single WGM allows direct, real-time tracking of the droplet size and refractive-index changes with a dynamic range that is not simply limited by the WGM linewidth. In this case, detection performance is actually not affected by amplitude noise but mostly by inherent laser-cavity frequency fluctuations. The feedback signal can be used to sense all such events occurring on a time scale within the locking loop bandwidth (10 kHz). For example, a droplet can probe nanoparticles or bacteria in a liquid sample and interact with them.

**Figure 11:**
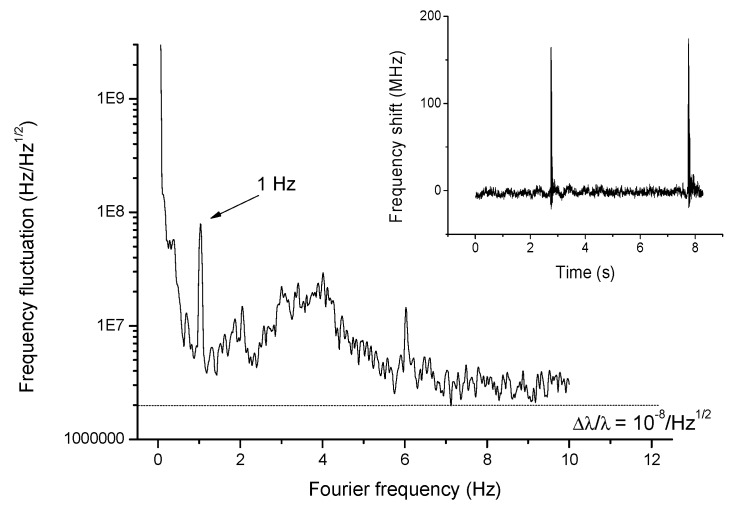
Real-time WGM shift measurements. Noise spectrum of the cavity-laser locking signal with application of a sinusoidal, slow mechanical perturbation. A wavelength-shift resolution limit is extrapolated from the noisefloor (dashed line). In the inset, the response of the droplet to a pulsed perturbation that mimics a particle binding to the surface is shown (All plots are reproduced with permission from [27]).

Alternatively, the targets may flow through a liquid channel, which is connected to the suspension wire or capillary, and merge to the droplet. In both cases, nanoparticles approaching the droplet would shift the WGM frequency as a consequence of the increased cavity size. As a test and calibration experiment, we applied a slow mechanical perturbation to the suspended droplet using the air flow generated by an electrostatic membrane placed just above the droplet. As shown in Figure 11, a wavelength resolution limit ranging from 10 to 20 fm/√Hz (i.e., Δλ/λ ≈ 10^−8^) can be obtained from the noise figure for particles with transit times ~1 s, i.e., on a Fourier frequency scale around 1 Hz. This noisefloor is mainly attributed to ambient disturbances, due to lack of insulation of the droplet. Despite that, the resolution of our set-up settles on the same level as that achieved with direct WGM shift observation in solid microresonators for virus detection [10]. In addition, the droplet sensor can detect fast, transient shift events as well. A step-like excitation is used to simulate the discrete shifts expected for adhesion of nanoparticles on the droplet surface, as shown in the inset of Figure 11. Considering the intrinsic sensitivity of our liquid resonator to external particles, no longer hampered by weak evanescent-field interaction, as opposed to solid microresonators, the droplet can be employed as a particle detector. The droplet resonance shifts with extremely high sensitivity according to the particle size and number. The dynamic range is not limited by the WGM linewidth, as opposed to most shift-based experiments, but it is only limited by the laser tunability range (~900 pm). The minimum detectable shift is in the order of Δλ/λ ≈ 10^−8^ resulting from ambient noise that adds fluctuations between the laser and the WGM resonance. The ultimate wavelength resolution is set by the free-running laser frequency noise that can be as low as 10^−10^ for a free-running diode laser [53], and can be pushed even further with active laser-stabilization systems.

## 4. Droplet Cavity Opto-Mechanics with Brillouin-Stimulated Surface Acoustic Waves

The interaction of electromagnetic radiation and mechanical motion has been extensively investigated in solid optical micro-resonators. Most of the experiments rest on the parametric excitation of vibrations in various structures, such as suspended micromirror cavities, microtoroids, microspheres, nanorods etc., exploiting radiation pressure as a coupling mechanism [54]. An alternative way consists of light scattering by a travelling sound wave [55], as originally suggested by Brillouin in 1922 [56], and later demonstrated in lasers under the form of stimulated Brillouin scattering (SBS) [57]. Basically, SBS in dielectrics can be driven by the refractive-index modulation induced in the material by the light field itself. Following the interaction with an acoustic wavefront, moving away from or towards the incident optical wave, the frequency of the scattered light is shifted downward [58,59] or upward [60] thereby generating a Stokes or an anti-Stokes sideband, respectively. The beating between the laser and Stokes fields contains a component at their difference frequency, i.e., at the sound frequency. The material response to this interference pattern, due to electrostriction or absorption, may in turn stimulate a sound wave that reinforces the Stokes wave, and so forth. If energy and momentum are conserved, this positive feedback becomes self-sustained giving an exponential growth of the Stokes sideband. If this occurs in a high-Q microresonator, where pump, Stokes and acoustic waves are all simultaneously resonant, a strong amplification of the vibrations takes place with energy transfer to the mechanical oscillator [59,60]. Momentum conservation (i.e., phase matching) also suggests that a reversal of the scattering direction from backward to forward allows accessing vibrations rates in the order of 50–100 MHz [59], much lower than the typical Brillouin values (~GHz), thus benefiting from a reduced material dissipation in the ultrasonic–hypersonic range [55].

In a recent paper, we experimentally demonstrated optical amplification of hypersonic waves circumferentially resonating in a stable droplet [61]. Co-resonating acoustic- and electromagnetic whispering-gallery modes that interact to exchange energy and momentum within the liquid cavity via stimulated Brillouin scattering are involved, thus enabling an optofluidic ultrasound– hypersound laser. The microresonators are obtained by immersion of a bare silica optical fiber in a pure sample of Xiameter^®^ PMX-200 silicone oil, spontaneously forming droplets with diameters between 100 μm and 1000 μm. The excitation and interrogation light source is a temperature-stabilized diode laser emitting around a wavelength of 640 nm. As already illustrated above, high-Q whispering-gallery modes are excited by focusing the free-space red beam tangential to the droplet surface with maximum power of about 8 mW. The effectively coupled optical power was approximately 6% of the incident one. The beam transmitted just after the droplet is detected by a fast photodetector (10-GHz electrical bandwidth) to interrogate optical and acoustic resonances.

Let us consider a pump optical wave at frequency *f_l_* and a travelling acoustic wave resonantly propagating along the droplet equator at a frequency *f_a_* satisfying the equation

2*πR* = *m_a_*⋅*V*/*f_a_* (10)

where *V* is the sound velocity in the liquid and *m_a_* is an integer equal to the number of acoustic wavelengths along the circumference. This mechanical mode creates an optical grating that photo-elastically scatters the pump beam: since the mechanical wave recedes with velocity *V*, it Doppler red-shifts the scattered light generating a Stokes wave at frequency

*f_s_* = *f_l_* − *f_a_* (11)

If the Stokes frequency falls into a second WGM resonance, its beat with the pump can in turn excite a vibration at *f_a_* in the cavity medium by electrostriction. If this optically-induced modulation also travels at the speed of sound, the whole process is self-sustained: then the Stokes wave undergoes a significant amplification that leads to a Brillouin laser. This phenomenological description is formalized by imposing energy and momentum conservation in the coupled-wave equation for the mechanical and optical waves. Its solution indicates that in this process the propagation constant of the pump, *β_l_*, must equal the sum of the propagation constants of the Stokes and acoustic modes, *β_s_* and *β_a_*. SBS in high-Q micro-resonators is thus possible when a triply-resonant condition is satisfied within the cavity for the pump, Stokes and acoustic waves. This task can be accomplished in multiple-mode resonators if the inter-modal distance equals the pump-Stokes frequency difference, which is made easier by the ability to change the optical WGM spatial distribution and transverse order [61,62].

Actually, phase matching may still be achieved if the pump photons and the scattered photons both populate the same optical resonance to a good extent [58,63]. For silicone oil droplets, this is feasible since the absorption-limited Q is between 10^6^ and 10^7^, and thus WGMs with different azimuthal and radial orders may coexist within the same resonance (linewidths ~50–500 MHz [35]). Their simultaneous excitation is facilitated by the free-space illumination scheme. Coupling to the micro-cavity exploits surface scattering that provides ample margins on WGMs’ spatial configuration. On the other hand, from momentum conservation, two interacting WGMs must exhibit azimuthal mode numbers where *m_s_* − *m_l_* = *m_a_*, with *m_a_* given by Equation (10). Expressing the phase-matching condition in terms of the WGM wavenumbers for a spherical micro-resonator, one obtains an analytical relation for the azimuthal mode number and the radial numbers (i.e., the qth zeros of the Airy function) in terms of the acoustic angular momentum number [61], which suggests that the coupling between the two neighboring optical modes can be optimized acting on their transverse orders.

**Figure 12:**
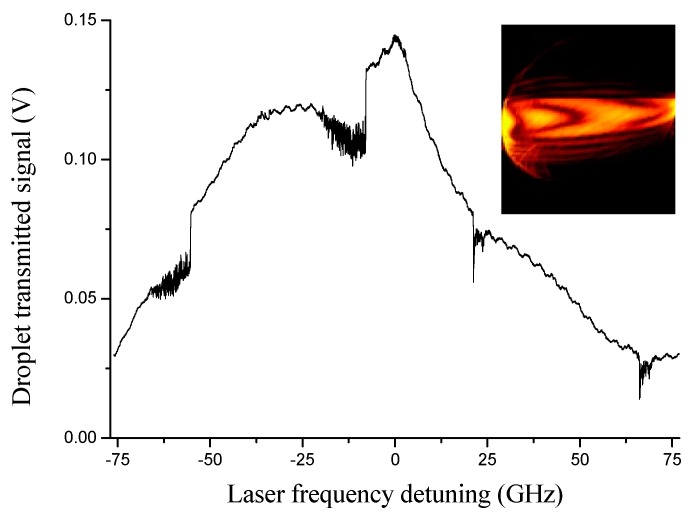
Optical resonances are shown as observed on the transmission of a 140-μm radius droplet along an ascending and descending wavelength scan, from left to right, respectively. Fast oscillations and broadening effects are observed on the resonances. In the inset, a camera image of the out-scattered radiation when the laser is resonant with the strongest mode (Reproduced with permission from [61]).

**Figure 13:**
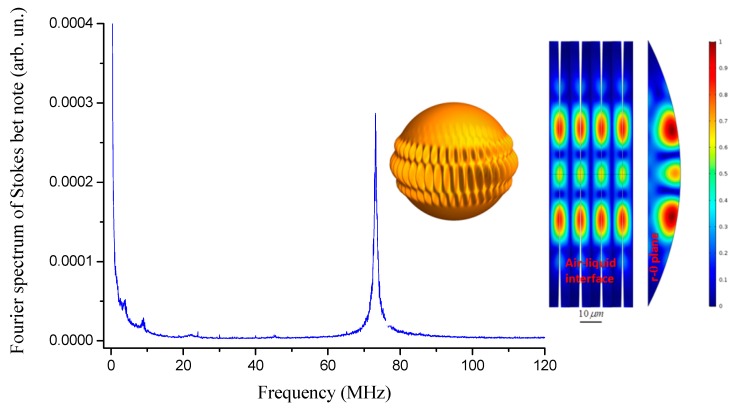
**Left**: FFT of the droplet transmission on resonance. The spectrum shows a large oscillations centered at 70 MHz (pump power of 360 μW). **Right**: Finite element method (FEM) simulation. A surface acoustic mode with high transverse order is shown in a 3-D artistic illustration (yellow sphere) with purposely exaggerated modulation depth along with its absolute displacement fields (scale on color bar) at liquid-phase boundary (*θ*-*φ* plane) and at radial-polar plane (*r*-*θ*) (All plots are reproduced with permission from [61]).

In order to interrogate the droplet oscillator, the laser is wavelength swept to excite WGMs. When the equator length is an integer multiple of the wavelength, an optical resonance shows up and a fraction of the incident power is coupled to circumferentially resonate into it. At the same time, light also undergoes strong scattering when the laser frequency is resonant. As shown in Figure 12, sharp dips appear on the droplet transmission with decreasing laser wavelength (increasing frequency). On the backward scan, instead, strongly broadened and asymmetric resonance WGM lineshapes manifest due to thermally-induced nonlinearities due to the intra-cavity build-up [35,61]. This effect leads to a self-locking mechanism that drives the laser in the vicinity of the resonance without the need for any active feedback system. In the inset of Figure 12, a camera recording of the out-scattered radiation when the laser is thermally self-locked to a strong WGM is shown: the deep intensity modulation indicates the presence of different equatorial resonances associated, as expected, with WGMs of different orders [64]. In this condition, the resonant transmitted power exhibits strong amplitude oscillations, in the MHz-range, which cannot be interpreted by the occurrence of droplet bulk acoustic modes [65]. Figure 13 shows the FFT of the transmission signal between 60 and 300 MHz. The spectrum contains distinct sharp features with the largest peak at 69.5 ± 0.8 MHz that is believed to originate from the beat between the pump laser and Brillouin forward-scattered modes [62].

A simulation was also carried out with a COMSOL^®^ code for a silicone oil droplet with radius of 140 μm. The resulting spatial distribution of the perturbation for a specific surface acoustic mode is shown in Figure 13 (color plot), pointing out the resonance mode around 70 MHz. This value is in good agreement with the first peak shown in the spectrum of Figure 13 thus confirming the whispering-gallery nature of the observed feature. The cavity transmission was also analyzed with a scanning Fabry–Pèrot interferometer with the laser on resonance, showing a new sideband oscillating at a frequency red-detuned by 70 MHz from the pump, which confirms the presence of the new optical component associated with the Stokes field, generated by cavity-enhanced forward SBS.

**Figure 14:**
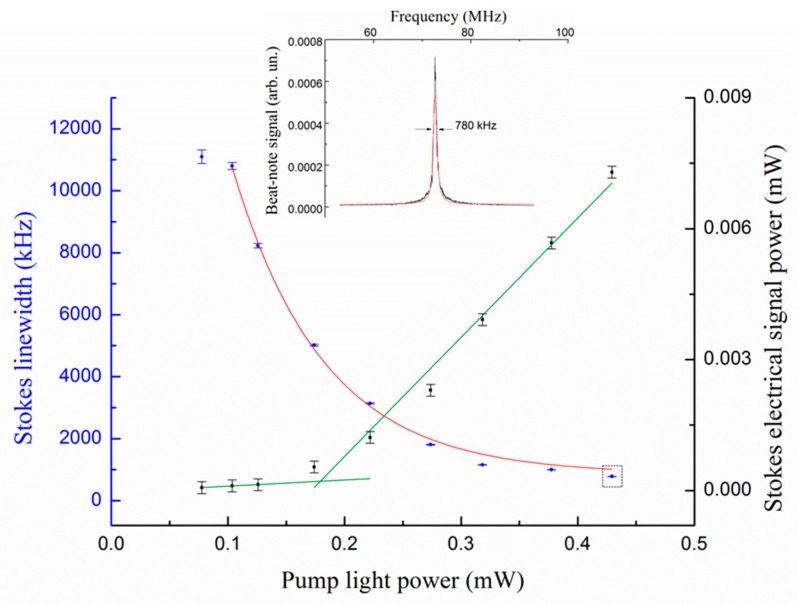
Stokes linewidth and power vs. pump light power injected into the droplet. The Stokes beat-note power (black squares) increases with pump and exhibits a knee with slope change at a threshold ~180 μW. The narrowest observed lineshape is shown in the inset with a red line representing a Lorentzian fit (All plots are reproduced with permission from [61]).

Figure 14 illustrates how the intra-cavity stimulated SBS process enhances a droplet surface wave and increases its quality factor. The beat-note power grows exponentially with pump power exhibiting a threshold at ~180 μW. This corresponds to a transition between the starting condition where Brownian noise is dominant and a new regime whereby the surface vibration is optically excited until the Brillouin gain provides its amplification and eventually overcomes material losses. Indeed, the beat-note, starting from an intrinsic mechanical quality factor Q_a_ = 5, is increased to an effective mechanical quality factor Q_a_* = 90.

It is interesting to note that the vibrating droplet behaves as a highly-coherent mechanical oscillator, i.e., a stable and narrow-linewidth liquid phonon laser at hypersonic frequency [66]. Similarly, other groups have successfully excited vibrations via optical whispering-gallery modes in liquid droplets of the same type [65,67]. In this case, the set-ups were based on near-infrared lasers coupled to droplet microresonators through an ultra-narrow tapered fiber while the observed mechanical resonances correspond to capillary waves around the kHz frequency range and bulk modes oscillating up to MHz, respectively.

## 5. Conclusions and Outlook

This review shows the big potential of droplet whispering-gallery mode microresonators as a sensitive and robust optical scheme for optical sensing in the real world. In particular, this approach provides great advantages for characterization of ultra-low absorbing liquid compounds and detection of biomolecules with ultra-low concentration in nanolitre volumes, where conventional methods are not easily applicable (e.g., water in the visible wavelength range). This would open a new route towards lab-in-a-droplet platforms aimed at spectroscopy and bio-sensing in the liquid phase. Working in a liquid device carries great advantages in chemical analysis as compared with solid microresonator sensors, which instead have several shortcomings. For instance, in conventional dielectric microresonators the major part of the WGM energy is trapped inside it, implying that analytes can only interact with the relatively weak evanescent tail penetrating in the external medium. As a consequence, the magnitude of induced WGM effects (resonance shifts, splitting or finesse change) are comparatively small. Furthermore, delivery of particles to the sensing region frequently relies on thermal diffusion, with long measurement times. Last but not least, surface binding or surface equilibrium conditions are required to measure concentrations of molecular species. Using a liquid droplet as an optical microresonator offers a number of benefits in this regard. First, the analytes can be easily incorporated into the droplet material, so that the liquid droplet serves as cavity-enhanced sensor and sample at the same time. In this way, they interact with the stronger portion of WGM, i.e., the intra-cavity field, thus leading to higher detection sensitivity and shorter measurement times. On the other hand, droplet resonators can be easily created without complex procedures, just relying on natural surface tension that gives the resonator a nearly-ideal spherical shape and surface quality. We have shown that, using a very simple set-up based on a pendant droplet and a free-space laser beam, optical-frequency locking to a WGM resonance and refractometric/spectroscopic detection are feasible [27,42]. In principle, at wavelengths where liquid absorption is negligible (e.g., water in the visible), surface scattering can be the only loss factor, leading to droplets with Q factors comparable to their solid counterpart (>10^8^) [35]. Importantly, liquid droplet cavities lend themselves to integration with microfluidic delivery and analysis platforms, particularly when combined with free space coupling schemes.

Recently, cavity opto-mechanics experiments gave evidence that sound and light waves can be mixed within a self-contained device entirely made of liquid, i.e., a single droplet [61,65]. Through radiation pressure exerted by resonant optical modes and stimulated Brillouin scattering, mechanical ‘bulk’ modes [65], capillary waves [67], and whispering-gallery waves equatorially propagating at the liquid-air boundary [61] were observed. All this suggests that a droplet can be simultaneously used as an optical and acoustic microresonator, wherein any optically-induced mechanical effect is stronger than in solids thanks to its softness. Uniquely, a real optofluidic hypersound-laser oscillating along the external surface around ~100 MHz was demonstrated using resonantly-coupled laser light [66]. These are the first, important steps towards the idea of simple droplets as fluid-dynamic laboratories on a miniaturized scale. As a future prospect, investigations on Brownian motion, viscoelasticity, hydrodynamic simulation, droplet coalescence, and surface capillarity phenomena in a controlled environment are also envisaged.
